# Modeling of ionic liquids viscosity via advanced white-box machine learning

**DOI:** 10.1038/s41598-024-55147-w

**Published:** 2024-04-15

**Authors:** Sajad Kiani, Fahimeh Hadavimoghaddam, Saeid Atashrouz, Dragutin Nedeljkovic, Abdolhossein Hemmati-Sarapardeh, Ahmad Mohaddespour

**Affiliations:** 1 Faculty of Science and Engineering, Swansea University, Swansea, SA1 8EN UK; 2 Key Laboratory of Continental Shale Hydrocarbon Accumulation and Efficient Development (Northeast Petroleum University), Ministry of Education, Northeast Petroleum University, Daqing, 163318 Heilongjiang China; 3 Institute of Unconventional Oil & Gas, Northeast Petroleum University, Daqing, 163318 China; 4 Department of Chemical Engineering, Amirkabir University of Technology (Tehran Polytechnic), Tehran, Iran; 5 College of Engineering and Technology, American University of the Middle East, Egaila, 54200 Kuwait; 6 Department of Petroleum Engineering, Shahid Bahonar University of Kerman, Kerman, Iran; 7 College of Construction Engineering, Jilin University, Changchun, China; 8 Department of Chemical Engineering, McGill University, Montreal, QC H3A 0C5 Canada

**Keywords:** Ionic liquids, Viscosity, GMDH, GP, White-box modeling, Leverage method, Chemical engineering, Environmental sciences

## Abstract

Ionic liquids (ILs) are more widely used within the industry than ever before, and accurate models of their physicochemical characteristics are becoming increasingly important during the process optimization. It is especially challenging to simulate the viscosity of ILs since there is no widely agreed explanation of how viscosity is determined in liquids. In this research, genetic programming (GP) and group method of data handling (GMDH) models were used as white-box machine learning approaches to predict the viscosity of pure ILs. These methods were developed based on a large open literature database of 2813 experimental viscosity values from 45 various ILs at different pressures (0.06–298.9 MPa) and temperatures (253.15–573 K). The models were developed based on five, six, and seven inputs, and it was found that all the models with seven inputs provided more accurate results, while the models with five and six inputs had acceptable accuracy and simpler formulas. Based on GMDH and GP proposed approaches, the suggested GMDH model with seven inputs gave the most exact results with an average absolute relative deviation (AARD) of 8.14% and a coefficient of determination (R^2^) of 0.98. The proposed techniques were compared with theoretical and empirical models available in the literature, and it was displayed that the GMDH model with seven inputs strongly outperforms the existing approaches. The leverage statistical analysis revealed that most of the experimental data were located within the applicability domains of both GMDH and GP models and were of high quality. Trend analysis also illustrated that the GMDH and GP models could follow the expected trends of viscosity with variations in pressure and temperature. In addition, the relevancy factor portrayed that the temperature had the greatest impact on the ILs viscosity. The findings of this study illustrated that the proposed models represented strong alternatives to time-consuming and costly experimental methods of ILs viscosity measurement.

## Introduction

Ionic Liquids (ILs) are novel, highly tunable, and unique compounds that emerged in response to interest in green chemical technologies^1^. In the last few decades, an enormous amount of research has gone into developing ILs for a wide range of uses, from industrial to molecular, such as gas absorption, energy storage, biotechnology, electrochemistry, separation, and fluid flow in porous media^2–5^. ILs are materials composed only of ions and having a melting point of less than 100 °C. They are created chemically when organic cations (such pyridinium, phosphonium, imidazolium, and ammonium) combine with organic and inorganic anions (like phosphates, halides, and sulfates)^5,6^. When Paul Walden originally described the IL (ethylammonium nitrate ([NHHH_2_] [NO_3_]) back in 1914, he had no idea that nearly a century later the field of ILs would become very significant^6^. Since 1996, the number of scientific papers on ILs has skyrocketed from just a few to over 8000 by 2020, far outpacing the growth rates of other well-known scientific fields^7^.

The type and arrangement of cations and anions, as well as the quantity of branching chains inside the molecules, are strongly linked to the characteristics of ILs^8^. ILs have several noteworthy characteristics, such as strong ion conductivity, remarkable permittivity, outstanding electrical properties, nonflammability, high heat capacity, and thermal and chemical durability^8,9^. Decades of research have led to the development of novel ionic liquids that can be synthesized to customize their physical and chemical characteristics for specific applications. As more ion combinations were developed, it became essential to describe their physical and chemical characteristics^10,11^. The addition of even small quantities of chemical precursors, for instance halides or water, might cause ILs to become very sensitive. Therefore, studying their physicochemical properties is vital. Density, electrical/thermal conductivity, sound speed, surface and interface properties, refractive index, and viscosity are necessary variables that require precise prediction and optimization. Viscosity is one of the key physicochemical characteristics that assist in assessing the purity, fluid dynamics, and intermolecular forces of ILs^11,12^. ILs have complex thermodynamic and physicochemical properties, therefore, modeling approaches and large datasets are needed to predict their viscosity. ILs have a viscosity range of between 10 and 10,000 mPa s, and their viscosity is much higher than conventional solvents (0.1–100 mPa s), which may be a big issue for applications requiring mass or charge transfer^13^. In this regard, accurate models of ILs viscosity are required for process modeling, which allow to minimize costs/energy and predict physicochemical properties of ILs^14–16^. Several computational methods, including group contribution methods (GCM), intelligent approaches (IA), and quantitative structure-property relationships (QSPR), can be used to determine the viscosity of ILs^15,17,18^. For example, Gardas and Coutinho used GCM to estimate the viscosity of ILs across a large temperature range (293–393 K) utilizing 500 data points from 29 distinct ILs (based on imidazolium, pyrrolidinium, and pyridinium). According to the results, 7.7% was the absolute average relative deviation (AARD) for determining the viscosity of ILs^19^. Other research was conducted by Gharagheizi et al. In this study, the viscosity of the IL was estimated using a GCM method. The model was based on 443 distinct ILs (1672 data points) with the temperature range from 253.15 to 433.15 K, and the result was an AARD of 6.3%^20^. Lazzús et al., in turn, developed a GCM-based linear model to predict ILs viscosity at temperatures ranging from 253 to 395 K, with an AARD of approximately 4.5%^21^. At the same time, AARD was about 11.4% in the study of Paduszynski et al. This work detailed the use of feed-forward neural network (FF-NN) based GCM using 13,000 data points (1484 ILs) with temperature and pressure ranges of 253–573 K and 0.06–350 MPa, respectively^22^. Finally, AARD for linear and nonlinear models were 10.68% and 6.58%, accordingly, as was suggested by the QSPR model of Zhao et al.^23^. This paper was based on a databank consisting of 1502 experimental points (89 ILs) across a broad range of temperatures (253.15–395.2 K) and pressures (0.1–300 MPa).

The nodes and layers of an artificial neural network (ANN) are controlled by a vast collection of equations. Aside from that, the number of nodes and levels in the network are decided either manually or at random^24,25^. The use of machine learning methods to model complicated systems has gained popularity recently^25–31^. Machine learning methods fall into two categories: black and white-box methods. Black-box models such as neural networks or gradient boosting may be quite accurate^26^. Black-box models (e.g., support vector regression (SVR) and decision tree) rely on a complicated computer-aided process, whereas white-box models (e.g., gene expression programming (GEP) and group method of data handling (GMDH)) clearly provide a simple and explainable approach^26,32–34^. Because white-box models provide a model that is more like to human language, they are often understandable to experts in practical applications. White-box models are based on patterns, rules, or decision trees^32,35^. The GMDH methodology, a self-organizing neural network, can not only describe the system's genome using simple polynomials, but it can also employ standard minimization procedures to determine the optimal configuration^24^. In our previous research, we used several black-box machine learning approaches for modeling the viscosity of ILs. Also, we developed a simple correlation using a trial-and-error procedure. However, the proposed correlation was not accurate enough and could predict the data with an AARD of 28%, which is high for engineering practices^25^. Thus, developing a more accurate correlation with high accuracy using advanced correlative approaches such as GMDH and GP appears to be a preferable research direction.

This work models a vast set of 2813 experimental viscosity values from 45 distinct IL using GP and GMDH models with diverse inputs. Additionally, empirical and theoretical methods—such as Eyring's theory (ET)—are used to estimate the viscosity of pure ILs. To determine which approach is the most correct, the dependability of the models that are provided is assessed using both graphical and statistical criteria. The sensitivity analysis is also used to determine how different input factors affect viscosity in relation to one another. Lastly, the quality of the experimental data is assessed and the application domain of the suggested models is determined using the leverage technique.

## Data collection

A model can be more accurate and widely applicable the more data points it contains. In order to do this, 2813 experimental viscosity data from 45 ILs were gathered from open literature sources at varying pressures (0.06–298.9 MPa), temperatures (253.15–573 K), and viscosities (1.13–9667.6 MPa.s)^36–48^.

1 $$\eta =f\left(T,P,{M}_{w},{T}_{c},{T}_{b},{P}_{c},\omega ,{V}_{c}\right)$$

Recognizing the potential risks associated with open literature data, a thorough screening method was implemented. This process evaluates the quality and consistency of experimental data based on specific criteria. Rigorous analysis was applied to any data points that raised questions, with verification achieved through direct contact with the original authors or alternative sources. This scrupulous approach enhances the robustness of the analytical data, fortifying the conclusions drawn. Strict standards for experimental data from open literature sources significantly contribute to the reliability of the results, highlighting the commitment to data dependability.

The dataset was randomly split into training (80%) and test (20%) subsets, ensuring that the test set remains undisclosed during parameter adjustments for independence. The application of k-fold cross-validation to the training subset played a pivotal role in this investigation. This approach ensures that each observation in the dataset is included in both the training and validation sets. The deliberate use of 6 k-folds for all models was strategic, with the choice depending on the data size—striking a balance between avoiding excessive or insufficient fold sizes. The train data underwent random partitioning into 6 folds, where the model fitting involved K-1 folds (i.e., 5 folds), and validation was conducted using the remaining fold.

## Model development

### Using Eyring’s Theory (ET) to calculate Pure Viscosity

**Figure 1:**
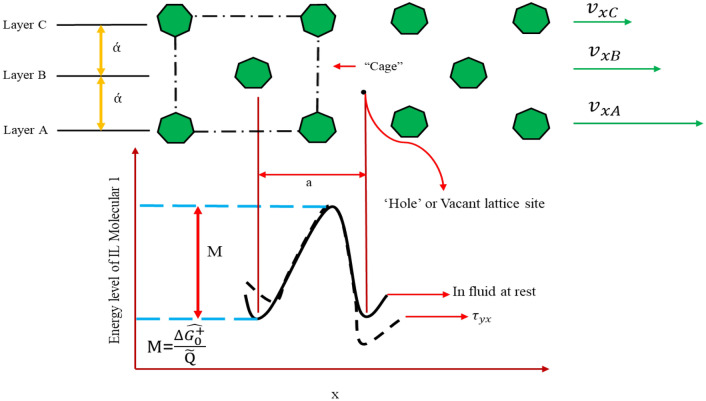
Illustration of a liquid flow's escape mechanism. Molecule 1 has to go through a "bottleneck" in order to get to the vacant position.^25^.

Kirkwood et al. have come up with a strong theory regarding how monatomic liquids transport^49^. This idea itself, however, does not provide immediate results. The absolute rate idea was suggested by Eyring et al.^50,51^. The individual molecules are always moving in a pure liquid at rest. But because the molecules are closely packed inside a "cage," the motion is mostly limited to the vibrations that each molecule generates in response to its nearest neighbors. The height-energy barrier $\frac{\Delta \widehat{{G}_{0}^{+}}}{{{\text{N}}}_{{\text{A}}}}$is this "cage" where ${{\text{N}}}_{{\text{A}}}$stands for the Avogadro number (molecules/g-mol). Additionally, in order to "escape" from the stationary fluid cage,  ${\Delta{G+0}}$ˆ, or a molar-free activation energy, is required. (Fig. 1)^25,51^.

Following Eyring’s theory (ET), a molecule escapes its “cage” into a resting liquid's “hole”^25^. As a result, every molecule moves in the length of “$\dot{\alpha }$” at a frequency “$f$”. The frequency is set by the rate expression:

2 $$f=\frac{KT}{P}{\text{exp}}(-\Delta \widehat{{G}^{+}} /TR)$$

where $K$, $P$, and $R$ are stand for the Boltzmann (J/K), the Planck constant, and the gas constant (J/mole·k), respectively. $T$ and $\Delta \widehat{{G}_{0}^{+}}$ represent the molar activation energy and absolute temperature (K) of the fluid at rest. Additionally, a fluid traveling in the x-direction with a gradient of velocity $\left(\frac{d{V}_{x}}{dy}\right)$experiences molecular reconfigurations more often. The potential energy barrier, deformed by the applied stress ${\tau }_{yx}$ is seen in Fig. 1 and will be expressed using the subsequent equation: 

3 $$-\Delta \widehat{{G}^{+}}=\widehat{\Delta {G}_{0}^{+}}\pm (\gamma /\dot{\alpha })\left(\frac{{\tau }_{yx}\widetilde{Q}}{2}\right)$$

where an estimate of how much work was performed on the molecules is shown by $\pm (\gamma /\dot{\alpha })\left(\frac{{\tau }_{yx}\widetilde{Q}}{2}\right)$. This is the mole liquid volume denoted by $\widetilde{Q}$. Equations (2) and (3) are then merged as follows:

4 $$f=\frac{KT}{p}{\text{exp}}\left(-\frac{\Delta \widehat{{G}_{0}^{+}}}{TR}\right){\text{exp}}\left(\frac{\pm \gamma {T}_{yx}\widetilde{Q}}{2\dot{\alpha }TR}\right)$$

The net velocity (Fig. 1) shows the separation between molecules in layer "A" and layer "B." The computation involves multiplying the net frequency of advancing jumps (${f}_{+}-{f}_{-}$) by the distance travel in each jump (${\dot{\alpha}}$). The frequency of forward and backward leaps are denotedby  "${f}_{+}$" and "${f}_{-}$". The following equation is used:

5 $${f}_{xA}-{f}_{xB}=\left({f}_{+}-{f}_{-}\right)\omega$$

Over a fairly small distance “$\dot{\alpha }$” between the two layers, a linear velocity profile may be observed, allowing:

6 $$-\frac{{\text{d}}{v}_{x}}{{\text{d}}y}=(\gamma /\dot{\alpha })\left(-{f}_{-}+{f}_{+}\right)$$

To sum up, Eqs. (4) and (6) are combined to form the following equation:

7 $$-\frac{{\text{d}}{v}_{x}}{{\text{d}}y}=(\gamma /\dot{\alpha })\left(\frac{KT}{p}{\text{exp}}\left(-\frac{\Delta \widehat{{G}_{0}^{+}}}{TR}\right)\right)\left({\text{exp}}\left(\frac{+\gamma {\tau }_{yx}\overline{Q}}{2\dot{\alpha }TR }\right)-{\text{exp}}\left(\frac{-\gamma {\tau }_{yx}\overrightarrow{Q}}{2\dot{TR}}\right)\right.=\left(\frac{\gamma }{\dot{\alpha }}\right)\left(\frac{KT}{p}{\text{exp}}\left(-\frac{\Delta \widehat{{G}_{0}^{+}}}{TR}\right)\right)\left(2{\text{sinh}}\frac{\gamma {\tau }_{yx}\widetilde{Q}}{2\dot{\alpha }TR}\right)$$

If $\frac{\gamma {\tau }_{yx}\widetilde{Q}}{2\dot{\alpha }TR}\ll 1$, the Taylor series can also be applied. Finally, the viscosity is derived using the following equation:

8 $$\eta ={\left(\frac{\gamma }{\dot{\alpha }}\right)}^{2}{{\text{N}}}_{A}h/\overline{Q}\mathrm{exp }\left(\frac{\widehat{\Delta {G}_{0}^{+}}}{TR}\right)$$

The unity factor, $\frac{\gamma }{\dot{\alpha }}$, makes the equation without compromising accuracy, since $\widehat{\Delta {G}_{0}^{+}}$ is acquired empirically to ensure that the equation’s values match the experimental results. However, it is demonstrated that, for a given fluid, the estimated $\widehat{\Delta {G}_{0}^{+}}$ (free activation energies) are almost constant when  fitting Eq. (8) to experimental viscosity values. This translates to the  boiling point internal energy of vaporization $\left(\Delta {\widehat{U}}_{vap}=\Delta {H}_{{\text{vap}}}-\mathrm{RT\Delta }{Z}_{{\text{vap}}}\right)$, which is given by Eq. (9) as follows^63^:

9 $$\widehat{\Delta {G}_{0}^{+}}\approx 0.408\Delta {\widehat{U}}_{vap}$$

By using this empiricism and setting $\frac{\dot{\alpha }}{\gamma }=1$, Eq. (8) becomes as follows when empiricism is set at $\frac{\dot{\alpha }}{\gamma }=1$:

10 $$\eta ={{\text{N}}}_{A}p/\overline{Q}\mathrm{exp }\left(\frac{0.408\Delta {\widehat{U}}_{\text{vap }}}{TR}\right)$$

The following is an accurate estimate of the vaporization energy provided by the Trouton's rule at the typical boiling point:

11 $$\Delta {\widehat{U}}_{vap}\approx\Delta {\widehat{H}}_{vap}-{T}_{b}R\cong 9.4{T}_{b}R$$

Equation (10), when approximated, reads as follows:

12 $$\eta ={{\text{N}}}_{A}p/\overline{Q}\mathrm{exp }\left(\frac{\lambda {T}_{b}}{T}\right)$$

where $\eta$ indicates the expected viscosity (mPa·s) of pure ILs. ${{\text{N}}}_{A}$ and $p$, respectively, are the Avogadro number (mole^−1^) and the Plank constant (J·s). The $\overline{Q }$ represents the volume of a mole of liquid (m^3^ mole^−1^), ${T}_{b}$ and $T$ stands for the boiling temperature (K) and temperature (K), respectively. To promote the performance of Eq. (12), a “$\lambda$” term was added to Eq. (12) in Excel program for each IL in this study. This term is not constant; rather, it varies depending on ionic liquid. Empiricism $\eta =A{\text{exp}}(B/T)$is compatible with eqs. (10) and (12) and appears to be a popular and useful approach.  Viscosity decreases with temperature, according to the theory.

### Group method of data handling (GMDH)

Ivakhnenko's data-management approach for groups matches Darwin's natural choice concept^52^. By merging two independent variables, the system chooses the optimal polynomial terms. The approach generates a generic multinomial term at each stage. The vast relationship multinomial Volterra–Kolmogorov–Gabor (VKG) analyzes the entire network^52^:

13 $${y}_{i}=a+\sum_{i=1}^{{N}_{v}} {b}_{i}{x}_{i}+\sum_{i=1}^{{N}_{v}} {c}_{ij}{x}_{i}{x}_{j}+\cdots +\sum_{i=1}^{{N}_{v}} \sum_{j=1}^{{N}_{v}} \dots \sum_{k=1}^{{N}_{v}} {d}_{ij\dots k}{x}_{i}{x}_{j}\dots {x}_{k}$$

In the above equation, the count of independent variables in the experiment is denoted by ${N}_{v}$. From a set of measured data with *N* data points, a matrix can be generated. The measured results $\overrightarrow{{V}_{y}}=\left({y}_{1},{y}_{2},\dots ,{y}_{n}\right)$ are represented on the left-hand side of the matrix, while the independent variables $\overrightarrow{{V}_{n}}=\left({x}_{1},{x}_{2},\dots ,{x}_{n}\right)$ are represented on the right-hand side of the matrix. Both sides of the matrix are produced from the same set of data. When two independent variables are coupled, a quadratic polynomial $$\left(\begin{array}{c}{N}_{v}\\ 2\end{array}\right)$$ can be used to estimate the actual data. Using ${N}_{v}$ parameters, here is a formula for $$\left(\begin{array}{c}{N}_{v}\\ 2\end{array}\right):$$

14 $${z}_{i}^{{\text{GMDH}}}=a{A}_{i}+b{B}_{i}+c{A}_{i}{B}_{i}+d{A}_{i}^{2}+e{B}_{i}^{2}+f$$

The matrix of independent variables can here be built using the vector of new variables $\overrightarrow{{V}_{z}}=\left({z}_{1},{z}_{2},\dots ,{z}_{n}\right)$. To modify the parameters of equations, the least squares method is utilized (15). The objective is to maintain the square of the deviation from the actual data as small as possible in each column:

15 $${\delta }_{j}^{2}=\sum_{i=1}^{{N}_{t}} {\left[{y}_{i}-{z}_{i}^{{\text{GMDH}}}\right]}^{2}j=\mathrm{1,2},\dots ,\left(\begin{array}{c}{N}_{v}\\ 2\end{array}\right)$$

In the above equation, ${N}_{t}$ denotes the count of datasets used. The measured data is used to construct training and testing subsets. The proportion of training and testing subsets is chosen at random. Equations are derived using the training set of data (15). The ideal set of parameters $\left({z}_{i}\right)$. Variations from planned results must fulfill the following criteria, based on the predefined requirement:

16 $${\delta }_{j}^{2}=\sum_{i={N}_{t}+1}^{N} {\left[{y}_{i}-{z}_{i}^{{\text{GMDH}}}\right]}^{2}<\varepsilon j=\mathrm{1,2},\dots ,\left(\begin{array}{c}{N}_{v}\\ 2\end{array}\right)$$

here, $\varepsilon$ is an optional/random value. Just the z columns that meet the criteria are kept, whereas the ones that do not are deleted. The entire variation is preserved after each repetition and compared to the prior repetitions to check if the least variation has been achieved.

### Genetic programming (GP)

GP is a breakthrough in optimization computing that combines traditional genetic methods with symbolic improvement^53–55^. It is predicated on an approach called "tree representation." This form is incredibly flexible since trees may represent full models of industrial systems, mathematical formulae, or computer programs. Creating model structures like differential equations, kinetic ordering, and steady-state models is best accomplished with this approach^56,57^. To achieve great variation, GP first creates an initial population, which consists of randomly selected individuals (trees). A new generation is finally formed by the software, which evaluates the individuals, selects individuals for reproduction, creates new individuals by mutation, crossover, and direct reproduction^57^. Unlike other optimization techniques, symbolic improvement uses the architectural arrangement of many symbols to convey workable solutions (that is, vectors of real values).

## Model assessment

### Statistical criteria

The models' validity was tested using the determination coefficient (R^2^), standard deviation (SD), average absolute relative deviation (AARD%), average relative deviation percent (ARD%), and root mean square error (RMSE). Below are the statistical parameters:

Determination Coefficient (R^2^): R^2^ is a regression coefficient that shows the model’s accuracy. The model fits the data better if it is close to 1. R^2^s mathematical formula is as follows:

17 $${{\text{R}}}^{2}=\frac{\sum_{i=1}^{{N}_{p}} {\left({\eta }_{i}^{{\text{exp}}}-\overline{\eta }\right)}^{2}-\sum_{i=1}^{{N}_{p}} {\left({\eta }_{i}^{cal}-{\eta }_{i}^{{\text{exp}}}\right)}^{2}}{\sum_{i=1}^{{N}_{p}} {\left({\eta }_{i}^{{\text{exp}}}-\overline{\eta }\right)}^{2}}$$

Average Relative Deviation (ARD%): The relative deviation of the anticipated outcomes from the experimental data is determined using the ARD%:

18 $$\mathrm{ARD\%}=\frac{100}{{N}_{P}}\sum_{j=1} \left(\frac{{\eta }_{j}^{{\text{exp}}}-{\eta }_{j}^{est}}{{\eta }_{j}^{exp}}\right)$$

Positive and negative ARD (%) represents a model’s underestimate and overestimate, respectively.

Standard Deviation (SD): SD is a metric used to quantify the degree of dispersion of data around the central point. This has the following definition:

19 $${\text{SD}}={\left(\frac{1}{N-1}\sum_{j=1}^{{N}_{P}} {\left(\frac{{\eta }_{j}^{exp}-{\eta }_{j}^{est}}{{\eta }_{j}^{exp}}\right)}^{2}\right)}^\frac{1}{2}$$

Average Absolute Relative Deviation (AARD%): The relative absolute deviation is used to quantify the difference between the actual or real data and the projected or represented data. It is shown by the equation that follows:

20 $${\text{AARD}}(\mathrm{\%}):100\times \frac{\sum_{j=1}^{{N}_{P}} \left|\frac{{\eta }_{j}^{nep}-{\eta }_{j}^{est}}{{\eta }_{j}^{exp}}\right|}{{N}_{P}}$$

Root Mean Square Error (RMSE): The RMSE is a frequently used statistical analysis approach for estimating the discrepancies between experimental and expected values. It goes by the name:

21 $$\text{RMSE }=\sqrt{\frac{1}{{N}_{P}}\sum_{j=1}^{{N}_{P}} {\left({\eta }_{j}^{exp}-{\eta }_{j}^{est}\right)}^{2}}$$

When calculating the average IL viscosity using experimental/real data,the experimental/real viscosity (*η*) and the number of data points ${N}_{P}$ are represented by the variables “*est*”, and “*exp*”, respectively.

### Graphical assessment of the models

Several graphical plots were used in this research to further evaluate the suggested models and measure their predicted performances. Among the visualization plots are diagrams showing the cumulative frequency and error distribution. In order to measure the distribution of error around the zero line and to indicate whether the model has a tendency to make mistakes, the percentage of relative deviation is displayed against target/real values in the error distribution. The cross-plot displays the estimated/represented value of the model in relation to the experimental data. After that, a slope line with a 45° unit is constructed to connect the experimental and represented/predicted values. A more accurate model is indicated by more data points that are shown along this line. The bulk of approximations will be inside a standard error range if the cumulative frequency is calculated from the absolute relative error.

## Results and discussion

### Development of models

Using 2813 points of data (45 ionic liquids) collected from the literature, models were developed. Table 2‘s “Total” refers to the whole set of data (2813 data points) that were used for analysis and modeling in the current research. The database was split into training sets (which made up 80% of the overall dataset) and test sets (20% of the total dataset) at random. The 563 data points in the "testing" set were used to track over-fitting errors and the reliability of the built models. The "training" subset (2250 data sets) caused changes to the model's structure and tuning parameters. T, P, Mw, V_c_, T_b_, T_c_, P_c_ and w were the input parameters, and IL viscosity was the output (Table 1).

**Table 1:** Dataset statistics acquired in this work.

	T (K)	P (mPa)	Tc (K)	Pc (bar)	Tb (K)	W	Mw (g/mole)	Exp.Viscosity (MPa s)
Mean	325.62	24.45	1005.87	22.30	723.94	0.60	346.66	191.97
Std	30.92	37.94	280.58	10.05	173.84	0.28	88.09	442.93
Min	253.15	0.06	520.06	2.63	410.77	0.22	201.23	1.13
25%	303.15	0.10	736.99	16.02	586.74	0.34	279.08	25.81
50%	323.15	6.00	1038.70	20.98	712.68	0.53	340.29	64.00
75%	343.15	40.00	1269.93	27.65	862.44	0.87	419.37	177.00
Max	573.00	298.90	1534.60	57.61	1130.30	1.10	515.13	9667.59

To begin with, the GMDH method was used to build a new empirical correlation. The viscosity of ILs with 5, 6, and 7 inputs was found to be:

5 Inputs:

22 $$\begin{array}{ll} &\eta = -2.87789 + T*0.0143915 - T*{N}_{1}*0.00131975 - {T}^{2}*1.78556e-05 + {N}_{1}*1.51847 - N2^2*0.0215748 \\ &{N}_{1} = -0.0340483 + P*0.00199063 - P*{N}_{2}*0.000467152 - {P}^{2}*4.36465e-06 + {N}_{2}*1.00943 \\ & {N}_{2} = -0.00484172 + N9*0.535395 + {N}_{8}*{N}_{3}*1.92525 - {N}_{8}^{2}*1.11778 + {N}_{2}*0.4892 - {N}_{2}^{2}*0.814881 \\ & {N}_{3} = 0.00291747 + {N}_{5}*0.258276 + {N}_{5}*{N}_{4}*9.67431 - {N}_{4}^{2}*5.13243 + {N}_{4}*0.759826 - {N}_{4}^{2}*4.54534\\ & {N}_{4} = -0.265189 + {T}_{c}*0.00125932 +{T}_{c}*{N}_{8}*0.000298289 - {T}_{c}^{2}*8.18089e-07 + {N}_{6}*0.53522 + {N}_{6}^{2}*0.0574476 \\ &{N}_{5} = 0.408599 + {T}_{c}*0.000100432 + {T}_{c}*{N}_{7}*0.000381021 - {T}_{c}^{2}*2.89505e-07 + {N}_{7}*0.350992 + {N}_{7}^{2}*0.086781\\ & {N}_{6} = 0.40796 - {P}_{c}*0.0250195 + {P}_{c}*{N}_{7}*0.00115096 + {P}_{c}^{2}*0.000437153 + {N}_{7}*0.854595 + {N}_{7}^{2}*0.0297431\\ &{N}_{7} = 0.274614 + P*N11*0.0015094 + {P}^{2}*6.77239e-06 + {N}_{9}*0.605881 + {N}_{8}^{2}*0.100009\\ & {N}_{8} = 7.44079 - T*0.0412914 - T*{N}_{10}*0.00645033 + {T}^{2}*5.49978e-05 + {N}_{10}*3.16623\\ & {N}_{9} = 10.5667 - T*0.0467936 - T*w*0.00582271 + {T}^{2}*5.09471e-05 + w*4.83785 - {\left(w\right)}^{2}*1.60314\\ &{N}_{10} = 0.865852 +P*0.00233429 + {P}^{2}*4.66924e-06 + w*2.3876 - {\left(w\right)}^{2}*1.18929 \end {array}$$

The 6 Inputs:

23 $$\begin{array}{ll} &\eta = -3.49184 + T*0.0171992 - T*{N}_{1}*0.0016858 - {T}^{2}*2.10848e-05 + {N}_{1}*1.68321 - {N}_{1}^{2}*0.033313\\ & {N}_{1} = 0.0365592 - {N}_{5}*N3*5.74908 + {N}_{5}^{2}*2.80849 + {N}_{2}*0.958807 + {N}_{2}^{2}*2.94393\\ & {N}_{2} = -0.895957 + M*0.00539033 + M*{N}_{3}*0.000164955 - {\left(M\right)}^{2}*7.8437e-06 + {N}_{3}*0.960834\\ &{N}_{3} = 0.00291747 + {N}_{5}*0.258276 + {N}_{5}*{N}_{4}*9.67431 - {N}_{5}^{2}*5.13243 + {N}_{4}*0.759826 - {N}_{4}^{2}*4.54534\\ & {N}_{4} = -0.265189 + {T}_{c} *0.00125932 + {T}_{c}*{N}_{6}*0.000298289 - {T}_{c}^{2}*8.18089e-07 + {N}_{6}*0.53522 + {N}_{6}^{2}*0.0574476\\ & {N}_{5} = 0.408599 + {T}_{c}*0.000100432 + {T}_{c}*{N}_{7}*0.000381021 - {T}_{c}^{2}*2.89505e-07 + {N}_{7}*0.350992 + {N}_{7}^{2}*0.086781\\ &{N}_{6} = 0.40796 - {P}_{c}*0.0250195 +{P}_{c}*N8*0.00115096 + ({P}_{c}^{2}*0.000437153 + {N}_{7}*0.854595 + {N}_{7}^{2}*0.0297431\\ & {N}_{6} = 0.40796 - {P}_{c}*0.0250195 +{P}_{c}*N8*0.00115096 + ({P}_{c}^{2}*0.000437153 + {N}_{7}*0.854595 + {N}_{7}^{2}*0.0297431\\ &{N}_{7} = 0.274614 + P*{N}_{8}*0.0015094 + {P}^{2}*6.77239e-06 + {N}_{8}*0.605881 + {N}_{8}^{2}*0.100009\\ &{N}_{8} = 10.5667 - T*0.0467936 - T*w*0.00582271 + {T}^{2}*5.09471e-05 + w*4.83785 - {\left(w\right)}^{2}*1.60314\end {array}$$

7 Inputs:

24 $$\begin{array}{ll} &\eta = -0.0100846 - {N}_{7}*{N}_{1}*2.82949 + {N}_{7}^{2}*1.37207 + {N}_{1}*1.01023 + {N}_{1}^{2}*1.45152.\\&{N}_{1} = -0.0134572 + P*0.0021813 - P*{N}_{2}*0.000587268 - {P}^{2}*5.26484e-06 + {N}_{2}*0.982197 + {N}_{2}^{2}*0.00878339;\\& {N}_{2} = 0.17471 - {P}_{c} *0.0126706 + {P}_{c} *{N}_{3} *0.00161699 + P{c}^{2}*0.00019748 + {N}_{3}*0.959782;\\ &{N}_{3} = -0.502724 + M*0.00270637 - M*{N}_{4}*0.000181006 - {M}^{2}*3.44871e-06 + {N}_{5}*1.06462;\\ & {N}_{4} = 0.0538836 - w*0.420022 - w*{N}_{5}*0.0630102 + {w}^{2}*0.347037 + {N}_{5}*1.06542;\\ & {N}_{5} = -0.159217 + N11*0.558586 + N11*{N}_{6}*4.28637 - N{11}^{2}*2.45863 + {N}_{6}*0.681851 - {N}_{6}^{2}*1.90027;\\ & {N}_{6} = -0.629478 + N18*0.82026 + N18*{N}_{7}*0.147292 - N{18}^{2}*0.232333 + {N}_{7}*0.683362;\\ &{N}_{7} = 0.646958 - {T}_{b}*0.000378377 + {T}_{b}*{N}_{8}*0.000677083 - {T}_{b}^{2}*3.17028e-07 + {N}_{8}*0.236567 + {N}_{8}^{2}*0.089335;\\ & {N}_{8} = 0.274614 + P*{N}_{10}*0.0015094 + {P}^{2}*6.77239e-06 + {N}_{10}*0.605881 + {N}_{10}^{2}*0.100009;\\ &{N}_{9} = 7.44079 - T*0.0412914 - T*{N}_{16}*0.00645033 + {T}^{2}*5.49978e-05 + {N}_{16}*3.16623;\\&{N}_{10} = 10.5667 - T*0.0467936 - T*w*0.00582271 + {T}^{2}*5.09471e-05 + w*4.83785 - {w}^{2}*1.60314;\\& {N}_{11} = 0.865852 + P*0.00233429 + {P}^{2}*4.66924e-06 + w*2.3876 - {w}^{2}*1.18929;\\& {N}_{12} = 0.162458 - {P}_{c} *{T}_{b} *7.76995e-05 + {P}_{c}^{2}*0.000712479 + {T}_{b} *0.00695937 - {T}_{b}^{2}*4.56726e-06;\end {array}$$

Furthermore, the equations below proposed for 5, 6, and 7 inputs in GP model:

5 Input:

25 $$Log(\eta )=\left({c}_{17}\frac{\frac{{c}_{0}}{\left(\left(\frac{{c}_{1}}{{\text{ln}}({c}_{2}{T}_{c})}-\left({c}_{3}T- \frac{{c}_{4}}{{c}_{5}w}\right)\right)-\left({c}_{6}P+({c}_{7}+{c}_{8}P)\right)\right)}}{\left(\frac{{c}_{9} }{{\text{ln}}({c}_{10}{P}_{c})}-\left(\frac{({c}_{11}+{c}_{12}P)}{{c}_{13}}-\left({c}_{14}T- \frac{{c}_{15}}{{c}_{16}w }\right)\right)\right)}+{c}_{18}\right)$$

$${c}_{0}=12.339 ;{c}_{1}=0.352 ;{c}_{2}=0.25504 ;{c}_{3}=-0.46065 ;{c}_{4}=17.271;{c}_{5}=2.5053 ;{c}_{6}=0.47496 ;{c}_{7}= 12.343 ;{c}_{8}=-0.34241 ;{c}_{9}=12.343 ;{c}_{10}=0.80501 ;{c}_{11}=-8.0693 ;{c}_{12}= -0.34241 ;{c}_{13}=8.3702;{c}_{14}=-0.46065 ;{c}_{15}=13.934;{c}_{16}=2.5053 ;{c}_{17}=-5409.2 ;{c}_{18}=-1.09.$$

6 Input:

26 $$Log(\eta )=\left({c}_{11}\frac{({\text{exp}}\left({\text{exp}}\left({c}_{0}w\right)\right)+{c}_{1})}{({c}_{2}T+{c}_{3}{P}_{c})(\left(\left(\left(\left({c}_{4}-{c}_{5}\right)-{c}_{6}T\right)-{c}_{7}P\right)+{c}_{8}T\right)-\frac{{\text{exp}}\left({c}_{9}M\right)}{{c}_{10}w})}+{c}_{12}\right)$$

$${c}_{0}=0.26945 ;{c}_{1}=15.569 ;{c}_{2}=0.7941 ;{c}_{3}=0.5728 ;{c}_{4}=-12.046;{c}_{5}=14.544 ;{c}_{6}=0.2556 ;{c}_{7}= 0.3401 ;{c}_{8}=1.3188 ;{c}_{9}=0.26945 ;{c}_{10}=-0.043257 ;{c}_{11}=15270 ;{c}_{12}= -1.1226$$.

7 Input:

27 $$Log(\eta )=\left(\frac{{c}_{0}}{\left({\text{ln}}\left(\left(\left({c}_{1}{T}_{c}+\frac{{c}_{2}{T}_{c}}{{c}_{3}}\right){c}_{4}w+\left({c}_{5}T+\left({c}_{6}{T}_{b}-{c}_{7}{T}_{b}\right)\right)\right)\right)\left({\text{ln}}\left(\left(\left({c}_{8}{T}_{c}+{c}_{9}{T}_{c}{c}_{10}\right){c}_{11}M+\left({c}_{12}T+\left({c}_{13}{T}_{c}+{c}_{14}{P}_{c}\right)\right)\right)\right){c}_{15}-{c}_{16}T \right)+\left({\text{ln}}\left({c}_{17}T\right){c}_{18}+\left({\text{exp}}\left({\text{ln}}\left({c}_{19}T {c}_{20}\right)\right)-{c}_{21}P \right)\right)\right)}*{c}_{22}+{c}_{23}\right)$$

$${c}_{0}=14.019 ;{c}_{1}=2.0204 ;{c}_{2}=0.25903 ;{c}_{3}=2.8184 ;{c}_{4}=0.88752;{c}_{5}=1.5553 ;{c}_{6}=0.46073 ;{c}_{7}= 1.2408 ;{c}_{8}=2.0204 ;{c}_{9}=0.25903 ;{c}_{10}=2.8184 ;{c}_{11}=0.88752 ;{c}_{12}= 0.91528 ;{c}_{13}=2.02;{c}_{14}=1.8657 ;{c}_{15}=-3.4975;{c}_{16}=1.0567 ;{c}_{17}=1.3443 ;{c}_{18}=-3.4975 ;{c}_{19}=1.553;{c}_{20}=11.329;{c}_{21}=2.3349;{c}_{22}=1213.7;{c}_{23}=-3.6512$$.

The critical temperature and pressure values of the IL are denoted *Tc* and *Pc*, respectively.  There is also an acentric factor (w), temperature (T), and pressure (P), as well as IL molecular weight (Mw), critical volume (Vc), and IL boiling temperature (Tb). The other parameters are the adjustable correlation coefficients (Table 2).

**Table 2:** Calculated the statistical requirements for the developed correlations.

Statistical criteria		RMSE	SD	R^2^	AARPE, %
7 Input: T, P, T_c_, P_c_, T_b_, w, Mw					
GP	Train	115.62	0.317	0.864	24.378
	Test	246.78	0.331	0.723	24.833
	Total	226.66	0.328	0.798	24.742
GMDH	Train	53.54	0.116	0.987	9.171
	Test	83.46	0.198	0.947	13.269
	Total	65.69	0.136	0.979	8.144
6 Input: T, P, T_c_, P_c_, w, Mw					
GP	Train	161.96	0.319	0.884	24.593
	Test	284.10	0.375	0.588	27.674
	Total	224.16	0.362	0.699	26.056
GMDH	Train	94.490	0.152	0.959	10.581
	Test	93.975	0.205	0.909	15.977
	Total	94.095	0.164	0.955	11.662
5 Input: T, P, T_c_, P_c_, w					
GP	Train	214.27	0.309	0.663	23.824
	Test	272.36	0.481	0.5358	32.101
	Total	261.18	0.353	0.650	27.824
GMDH	Train	99.374	0.1146	0.955	8.0798
	Test	121.34	0.236	0.855	16.911
	Total	104.178	0.146	0.944	10.951

The RMSE, SD, R^2^, and AARPE% for the proposed correlation are calculated for the GP and GMDH models in Table 2.

**Figure 2:**
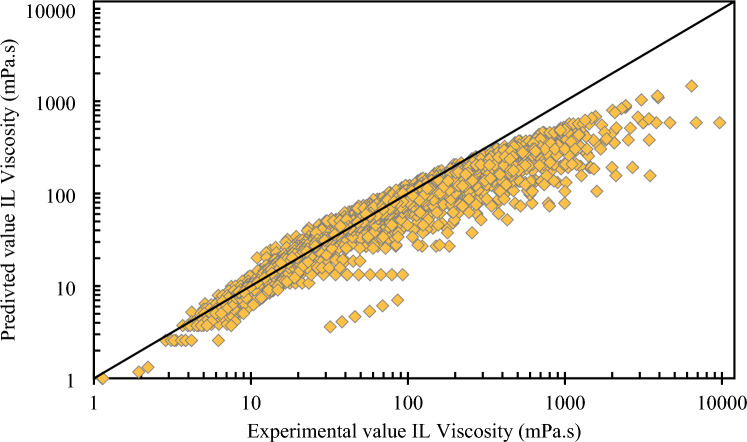
Cross plot of the proposed Eyring’s theory for viscosity of ILs.

The cross-plots on the results of the experimental viscosity data and the predicted data for the given correlation are displayed in Fig. 2. Around the unit-slope line, this figure shows a medium-uniform distribution of forecasts. The viscosity of the ILs that were taken from the database was estimated using temperature (T) and boiling temperature (Tb), in accordance with Eyring's theory (Eq. 13). AARD stands for 21.86%. The expected vs experimental IL viscosity is also plotted in a logarithmic cross-plot in Fig. 2. The data points were somewhat near the diagonal line, indicating moderate conformity. But data indicates that Arrhenius reliance does not match the experimental transport characteristics of ILs, which is why Eyring's theory does not hold up. In fact, ILs viscosity decreased as temperature rose, and this feature has to be changed by new model improvements. In order to define the thermal characteristics of ILs, the Vogel–Tamman–Fulcher (VTF) development is frequently used. This provides the basis for a complex energy landscape with several local potential energy minimums and a broad variety of energy barriers^58,59^.

The white-box machine learning models were carried out using GP and GMDH and compared to ET and Mousavi's model^25^. "White-box" models in machine learning are those that are easy for experts in the application area to understand. These models, in general, provide a fair mix between explainability and accuracy. The numerical assessment of the created methods is presented in Table 3. With the use of a GMDH optimizer, it was shown that the usage of seven inputs was the best design for forecasting the viscosity of ILs since it can anticipate the whole data collection with more accuracy than other approaches (AARD% = 8.14).

**Table 3:** Statistical comparison between the Eyring modified model correlations and new developed correlation model with various inputs.

	AARD (%)	RMSE	SD	R^2^
GP—5 inputs	27.82	261.18	0.35	0.65
GMDH—5 inputs	10.95	104.17	0.14	0.94
GP—6 inputs	26.05	224.16	0.36	0.70
GMDH—6 inputs	11.66	94.09	0.16	0.95
GP—7 inputs	24.74	226.66	0.32	0.79
GMDH—7 inputs	8.14	65.69	0.13	0.98
Eyring modified model	25.76	371.97	0.33	0.29
Mousavi et al. correlation	28.34	394.12	0.36	0.20

### Statistical evaluation

To illustrate the error margin, many statistical metrics were computed for both created models with different inputs, including ARD, AARD, RMSE, SD, and R^2^. As more input data was provided, the AARD, ARD, SD, and RMSE values for the test and training datasets decreased, as seen in Table 3 for the GP and GMDH models. . The following is a breakdown of the models based on how accurate they are: Mousavi et al. correlation < Eyring theory < GP < GMDH. As a result, the GMDH model may produce more reliable estimates than the other established models.

### Graphical error analysis

**Figure 3:**
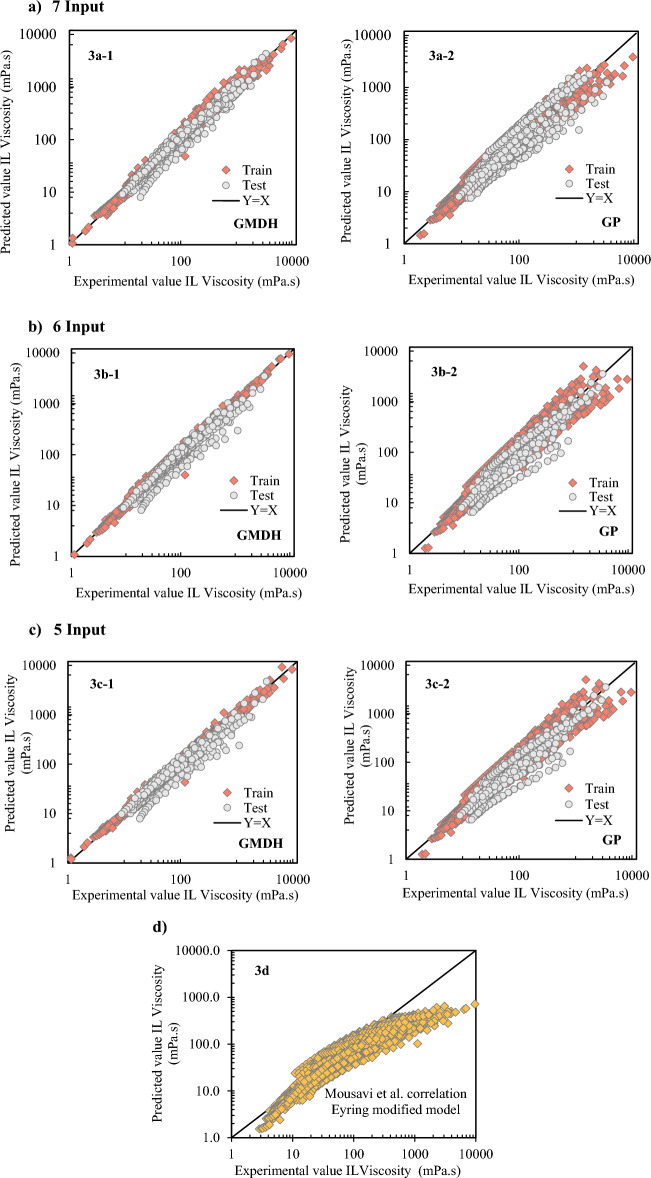
Comparison of the cross plots of the predicted correlations. Subfigures include: Fig 3(**a**) with 7 inputs (3a-1: GMDH, 3a-2: GP), Fig 3(**b**) with 5 inputs (3b-1: GMDH, 3b-2: GP), Fig 3(**c**) with 3 inputs (3c-1: GMDH, 3c-2: GP), and Fig 3(**d**) (modified Eyring model).

A number of graphical error evaluations developed from the GP and GMDH models were examined in order to offer a more lucid evaluation of the models' efficacy. To evaluate the models, the predicted viscosity measurements were compared to the experimental measurements shown in Fig. 3(a–c). For the GP and GMDH models, there is a high formation of points around the unit slope in both the test and training datasets. The observed viscosity values are shown to be more accurate by the GMDH correlations than by the GP and Mousavi et al. correlations (Fig. 3c). As indicated in Fig. 3, the data distribution for GMDH correlation with 7 inputs is more on the slop line than the GMDH data with 5 and 6 inputs. The GMDH decreases overall relative deviation, resulting in the smallest error margin.

**Figure 4:**
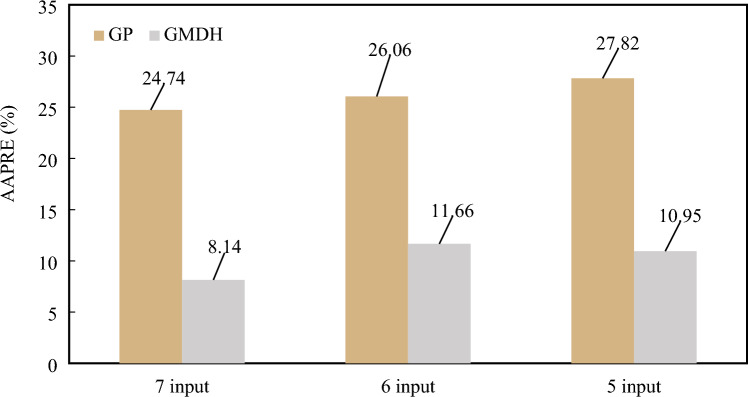
Comparison between the AARE values of the GMDH and GP models.

The AARE% of the white-box machine learning models is shown against the number of input parameters in Fig. 4. Comparing the GP model with experimental data indicated that it was less accurate, less flexible, and less well-suited than the GMDH model. Furthermore, the GMDH model with 7 inputs showed higher accuracy with experimental viscosity data in comparison to the GMDH trained on 5 and 6 inputs.

**Figure 5:**
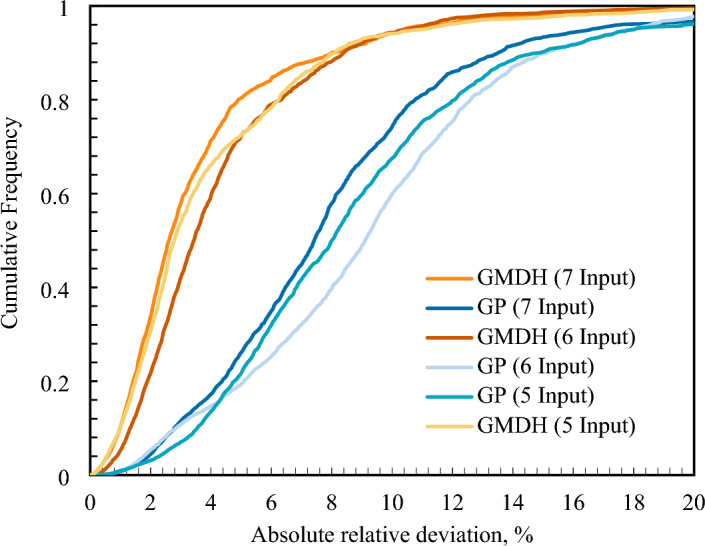
Absolute relative deviation cumulative frequency for various models based on GMDH and GP models.

To show the models' level of competence, comparative graphs are used, such as cumulative frequency plots. The GMDH has the maximum cumulative frequency for a given absolute relative deviation, as seen in Fig. 5. To put it another way, the GMDH model predicted almost 70% of the data points as we got closer to the ARD of less than 4%, but the corresponding values for the GP models were 9%, 11%, and 10%, respectively.

**Figure 6:**
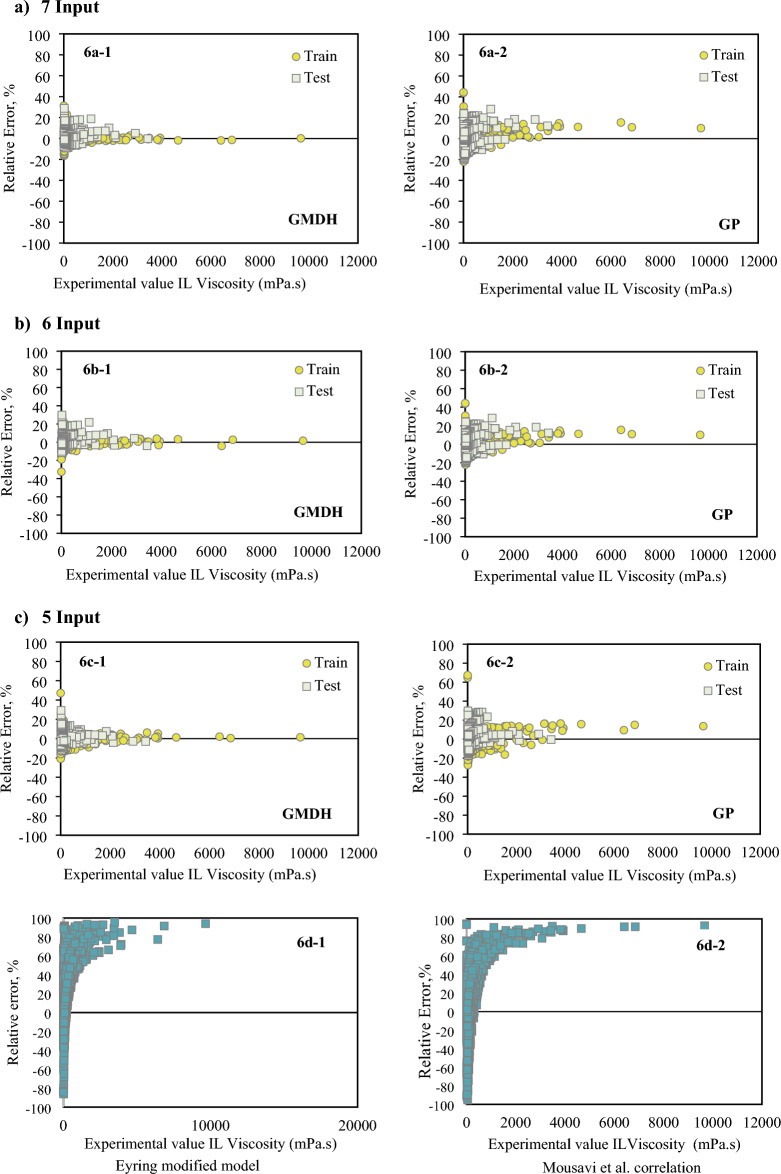
Error distribution plots of the developed correlations compared to the Eyring modified and Mousavi’s models. Fig 6(**a**) with 7 inputs (6a-1: GMDH, 6a-2: GP), Fig 6(**b**) with 6 inputs (6b-1: GMDH, 6b-2: GP), Fig 6(**c**) with 5 inputs (5c-1: GMDH, 5c-2: GP), and Fig 6(**d**) (6d-1: modified Eyring model, 6d-2: modified Mousavi model).

Figure 6 presents a comparison of the created models with respect to their relative deviations. The model's ability to precisely predict the viscosity of ILs is demonstrated by the dense cluster of dots surrounding the zero line. As can be seen, the GMDH model with 5, 6, and 7 inputs estimated viscosity better than GP, Eyring theory, and Mousavi et al. correlations.

**Figure 7:**
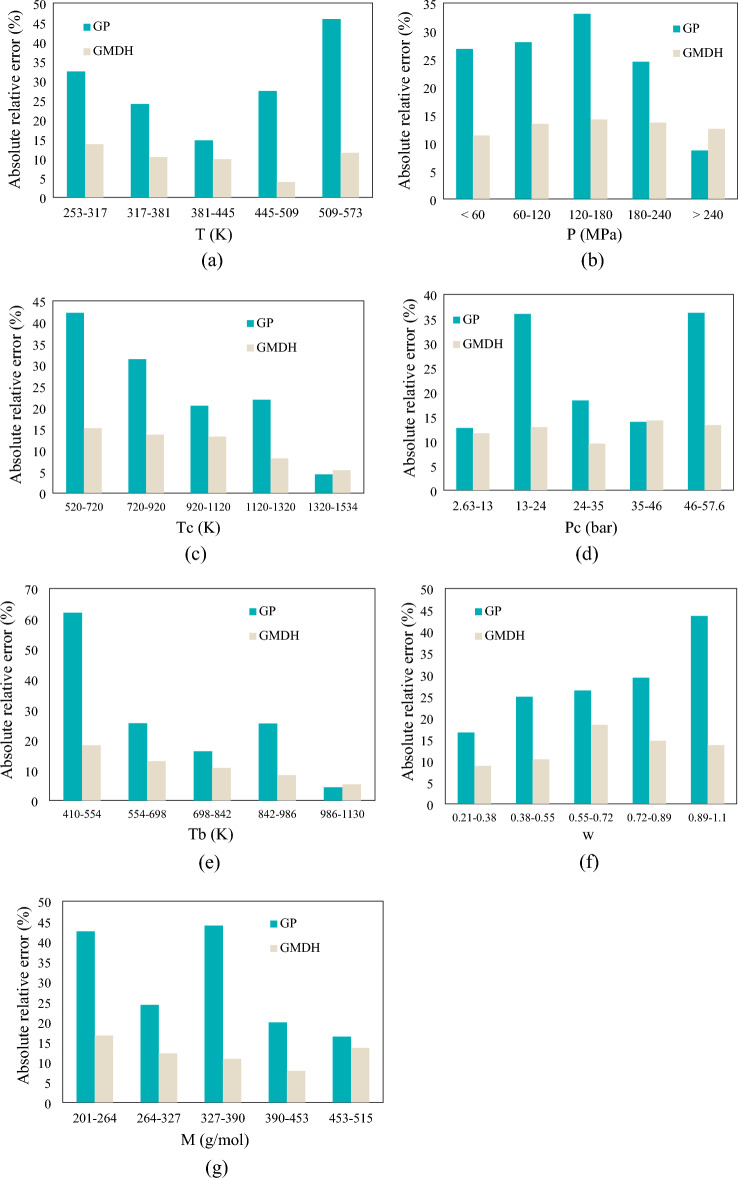
AARE for the correlations between the GMDH and GP correlations with 7 inputs. Temperature; pressure; critical temperature; critical pressure; boiling temperature; acentric factor; and molecular weight are represented by (**a**–**g**).

Figure 7 compares the acentric factor, molecular weight, boiling temperature, critical temperature / pressure, temperature, and pressure impacts (7 inputs) on AARD (%) for the GMDH and GP models under investigation. According to our findings, the GP model is more sensitive to changes, which leads to greater parameter values than the GMDH model, which was shown to be less susceptible. The GP model, for instance, is very temperature-sensitive (Fig. 7a). Thus, the GMDH model may be applied in a wide range of temperatures with a lower relative error of ARE <15%,  whereas the GP model can only be utilized in a narrow range of temperatures (381-445 K) with a minimum relative error of 14.6%.

**Figure 8:**
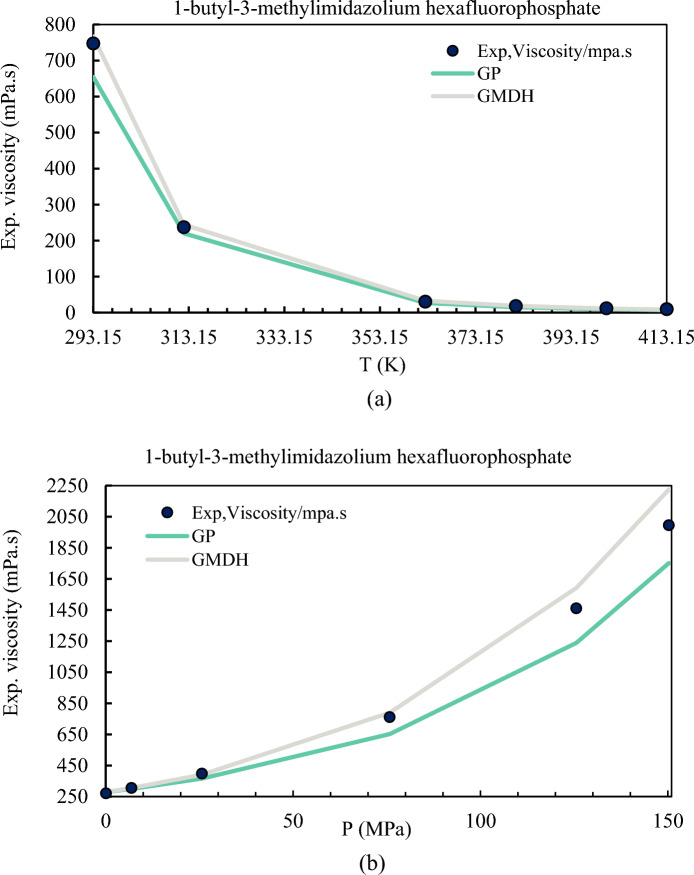
Correlation of the 1-butyl-3-methylimidazolium hexafluorophosphate viscosity for the generated correlation (7 input) with experimental data. (**a**) Viscosity-temperature; (**b**) Viscosity-pressure.

AARD values of 8.14% and 25.76% for the 7 inputs are displayed in Table 3 and are thus retained for future analyses since they are among the best responses for the GMDH and GP models. Based on the GMDH and GP correlations, Fig. 8 shows how temperature and pressure affect 1-ethyl-3-methylimidazolium hexafluorophosphate. The anticipated viscosity of ILs using both models is consistent with the experimental dataset, as expected. Viscosity assessments for ILs using GP correlations are, in turn, inconsistent, as seen in Fig. 8, and come with large error margins. As can be observed  in Fig. 8b, there is a physical link between the temperature and the GMDH model; but, as the pressure increases, neither model can adequately represent the experimental data.

### Identifying outliers in experimental data, GMDH, and GP models

Finding data that significantly differs from the bulk of the data in a database is the aim of outlier (or aberrant) identification^60,61^. Leverage is a well-known approach for doing this^60,62^. Standardized residuals (R) and the Hat matrix (H) are used^62^. The R value for each data point can be found using the below equation:

28 $${R}_{i}=\frac{{z}_{i}}{{\left(\mathit{MSE}\left(1-{H}_{ii}\right)\right)}^\frac{1}{2}}$$

$MSE$ stands for mean square error (MSE), while the ${i}_{th}$ data point's error and ${ii}_{th}$ Hat indices (Leverage) are represented by ${z}_{i}$ and ${H}_{ii}$^63^. In addition, the following formula may be used to calculate Hat index (or Leverage):^64^

29 $${\text{H}}={\text{X}}{\left({X}^{t}X\right)}^{-1}{X}^{t}$$

here, *X* represents a two-dimensional *q* × *w* matrix (where “*q*” shows the number of data and “*w*” is the count of input variables). Also, *X*^*t*^ is transpose of matrix. The outliers were investigated using the Williams plot after the *R* and *H* values were measured. In addition, the Leverage limit (H*), a parameter defined as 3*a/b*, where *b* stands for the count of data points and *a* is the number of model parameters plus one, is applied in this approach.

**Figure 9:**
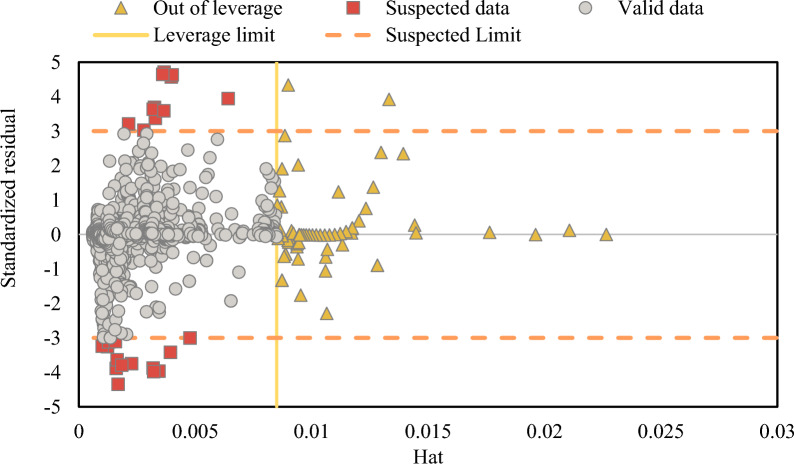
Williams plot for outlier the proposed GMDH.

The calculated R values must be within [− 3, + 3] standard deviations in order to encompass 99.7% of the normally distributed data^17,62^. The model is statistically valid if a significant proportion of data points are in the range of ${H}^{*}\ge H\ge 0$ and $3\ge R\ge -3$^17^. Since they are highly expected yet outside of the application domain, data points in the range of $-3\le R\le 3$ and ${H}^{*}\le H$ are  referred to as "Good High Leverage" points. Conversely, data points with *R* values larger than or less than -3 are referred to as "Bad High Leverage" data points. These regions are beyond the applicability range of the model and have significant levels of uncertainty. It is clear that reliable data significantly affect the GMDH (7 inputs) model's performance, making it the best model used in this study. The H* value, as per the suggested model, was 0.0085. The GMDH model's Williams plot is shown in Fig. 9 into the statistically significant range of  $0\le H\le 0.0085$ and $-3\le R\le 3$, all data points appear to fit into the established GMDH model. Less residual value normalization leads to an increase in reliability. However, However, 24 suspicious data points, or fewer than 1% of the total data in Fig. 9, either  $R$ <  − 3 or $R$ > 3, making them outliers with considerable uncertainty. Furthermore, 77 data points, or 3% of all data had *H* > 0.0085. These points are all in the range of $-3\le R\le 3$, which indicates that they are all Good High Leverage regardless of their Hat (Leverage) values.

### Variables' relative importance

**Figure 10:**
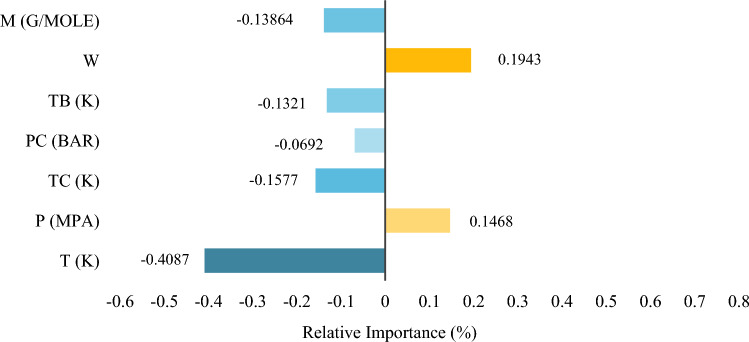
Evaluation of the input parameters' impact on ILs viscosity.

When taking the GMDH model, all input variables were tested to see how much of an influence they had on the viscosity of ILs. The relative significance of the inputs with respect to one another is shown in Fig. 10. One measure used to evaluate each input parameter's impact on the pure viscosity of ILs as a model output is the relevance factor (*r*). Negative values indicate an inverse correlation between the input and output parameters, and vice versa. Relevance Factor ($r$) values are analyzed in accordance with the following equation^65^:

30 $$r\left({I}_{i},\eta \right)=\frac{\sum_{j=1}^{n} \left({I}_{ij}-{\overline{I} }_{i}\right)\left({\eta }_{j}-\overline{\eta }\right)}{{\left(\sum_{j=1}^{n} {\left({I}_{i,j}-{\overline{I} }_{j}\right)}^{2}\sum_{j=1}^{n} {\left({\eta }_{j}-\overline{\eta }\right)}^{2}\right)}^{0.5}}$$

where $n$ represents the number of datasets. Also, the j-th value, and the mean of the *I*-th input are respectively represented by the variables,${I}_{i,j}$, and ${\overline{I} }_{i}$. Whereas $\overline{\eta }$ denotes the average value of the predicted ILs viscosity, while ${\eta }_{j}$ represnts the j-th value of the represented/expected viscosity. Based on the GMDH (as the output), Fig. 10 displays the relative effects of each parameter on the pure viscosity of ILs. It is demonstrated that temperature and the acentric factor significantly affect the model's output.

Viscosity increases with an increase in pressure or acentric factor in pure ionic liquids. As Fig. 10 illustrates, increasing $T,{M}_{w},{V}_{c},{T}_{b},{T}_{c}$, and ${P}_{c}$ parameters will result in a decrease in  the viscosity of ILs, since they have negative relevance factors. Moreover, the temperature has the most significant effect on the viscosity of ILs compared to other inputs.

We compared our models to a nonlinear artificial neural network (ANN) model using a dataset of 8,523 IL-water mixture viscosity data points^16^. The assessment included critical performance indicators such as mean absolute error (MAE) and R-squared (R^2^). The results show that the GP and GMDH models have equivalent, if not greater, prediction accuracy, with benefits in simplicity and interpretability. This comparative research not only supports our models' effectiveness but also highlights their potential as reliable methods for forecasting IL viscosity. Our target in this research was in line with the goal of obtaining better predictions about the physical features of ILs^15^. The main concern, though, is making accurate predictions regarding the viscosity of pure IL. We used the genetic programming (GP) and group method of data handling (GMDH) techniques to do this. In particular, our study adds custom models with clear benefits, focusing on accuracy and ease of use in determining the viscosity of pure ILs.

## Conclusions

The GMDH model was obtained by modeling 2813 experimental findings from 45 ILs based on temperature, pressure, molecular weight, critical volume, and acentric factor. Furthermore, IL viscosity was calculated using temperature and boiling temperature in accordance with Eyring's hypothesis. There were statistical and graphical comparisons between GMDH and experimental data in order to evaluate the model's efficacy. AARD, ARD, RMSE, and R^2^ parameters indicated that the GMDH model performed rather well. Using the relevance factor, the impact of input characteristics on the model's target parameter was also investigated. The relevance factor illustrated that the temperature is the most important parameter affecting ILs viscosity. Finally, the employed dataset's reliability and validity were assessed using the leverage statistics. In our case, Williams' plot was applied to study the established paradigm's applicability domain and data collection. Only a small number of data points were found to be outside the realm of applicability.  In light of all the above, the developed GMDH model is able to accurately forecast IL viscosity and obtain IL physicochemical parameters in different chemical engineering processes.

## Data Availability

All data have been gathered from the literature. All references used for extracting the required data have been cited in the text. However, the data will be available from the corresponding author upon reasonable request.
